# Urbanization, economic development, and environmental changes in transitional economies in the global south: a case of Yangon

**DOI:** 10.1186/s13717-022-00409-6

**Published:** 2022-11-11

**Authors:** Peilei Fan, Jiquan Chen, Cadi Fung, Zaw Naing, Zutao Ouyang, Khaing Moe Nyunt, Zin Nwe Myint, Jiaguo Qi, Joseph P. Messina, Soe W. Myint, Brad G. Peter

**Affiliations:** 1 School of Planning, Design, and Construction and Center for Global Change and Earth Observations, Michigan State University, East Lansing, MI 48824 USA; 2 Department of Geography, Environment, and Spatial Sciences and Center for Global Change and Earth Observations, Michigan State University, East Lansing, MI 48824 USA; 3 Department of Geography, University of Alabama, Tuscaloosa, AL 35487 USA; 4 Mandalay Technology, Yangon, Myanmar; 5 Earth System Science, Stanford University, Stanford, CA 94305 USA; 6 Yangon City Development Committee, Yangon, Myanmar; 7 Yangon University, Yangon, Myanmar; 8 College of Arts and Sciences, University of Alabama, Tuscaloosa, AL 35487 USA; 9 School of Geographical Sciences and Urban Planning, Arizona State University, Tempe, AZ 85287-5302 USA; 10 Department of Geosciences, University of Arkansas, Fayetteville, AR 72701P USA

**Keywords:** Urbanization, Economic development, Environmental change, Transitional economy, Globalization, Extreme climate event, Capital relocation, Myanmar

## Abstract

**Background:**

Transitional economies in Southeast Asia—a distinct group of developing countries—have experienced rapid urbanization in the past several decades due to the economic transition that fundamentally changed the function of their economies, societies and the environment. Myanmar, one of the least developed transitional economies in Southeast Asia, increased urbanization substantially from 25% in 1990 to 31% in 2019. However, major knowledge gaps exist in understanding the changes in urban land use and land cover and environment and their drivers in its cities.

**Methods:**

We studied Yangon, the largest city in Myanmar, for the urbanization, environmental changes, and the underlying driving forces in a radically transitioned economy in the developing world. Based on satellite imagery and historic land use maps, we quantified the expansion of urban built-up land and constructed the land conversion matrix from 1990 through 2020. We also used three air pollutants to illustrate the changes in environmental conditions. We analyzed the coupled dynamics among urbanization, economic development, and environmental changes. Through conducting a workshop with 20 local experts, we further analyzed the influence of human systems and natural systems on Yangon’s urbanization and sustainability.

**Results:**

The city of Yangon expanded urban built-up land rapidly from 1990 to 2000, slowed down from 2000 to 2010, but gained momentum again from 2010 to 2020, with most newly added urban built-up land appearing to be converted from farmland and green land in both 1990–2000 and 2010–2020. Furthermore, the air pollutant concentration of CO decreased, but that of NO_2_ and PM_2.5_ increased in recent years. A positive correlation exists between population and economic development and the concentration of PM_2.5_ is highly associated with population, the economy, and the number of vehicles. Finally, the expert panel also identified other potential drivers for urbanization, including the extreme climate event of Cyclone Nargis, capital relocation, and globalization.

**Conclusions:**

Our research highlights the dramatic expansion of urban land and degradation of urban environment measured by air pollutants and interdependent changes between urbanization, economic development, and environmental changes.

## Introduction

Urbanization has been viewed as one of the five ways that the world will change radically during the twenty-first century (Wolchover 2011), with developing countries being the main force to the increasingly urban world. Cohen (2006) estimated that virtually all population growth will be concentrated in urban areas of the developing world in the next three decades. Transitional economies in Southeast Asia—a distinct group of developing countries—have experienced rapid urbanization in the past several decades due to the economic transition that fundamentally changed the function of their economies, societies and the environment. Yet, there remain several major knowledge gaps in understanding on how state and market together have affected the urbanization process throughout the transition (Fan et al. 2017a, b; Fan 2022; Sýkora and Bouzarovski 2012), particularly on the changes in urban land cover and environment that had been directly and indirectly shaped by the multiple external drivers, such as extreme events, globalization, and institutional shifts (e.g., relocation of the capital city).

The Republic of the Union of Myanmar, for example, is one of the least developed transitional economies in Southeast Asia, albeit the urbanization ratio increased significantly since the early 1990s. Its urbanization was at 22% in 1968, 25% in 1990, and 31% in 2019 (World Bank 2021). This rapid urbanization, spurred by the country’s economic liberalization beginning in the late 1980s, has been accompanied by a dramatic societal change in the most recent decade, which has featured privatization and the democratic transition in the political sphere in 2011. Myanmar initiated economic liberalization in 1988, focusing on marketization of the agricultural sector by allowing state enterprises to operate as market-oriented firms. In addition to this marketization, foreign investment was permitted through the Foreign Investment Law in 1988 that encouraged the benefits of tax exemptions for 3 years (Rigg 2004). Liberalization was evaluated as having improved the living conditions of farmers with a rise in income and other available agricultural technologies (Okamoto 2007). However, critics argued that economic liberalization negatively affected the livelihood of the people. For example, environmental degradation has been coupled with resource extraction by foreign investors, elevated unemployment rates of farmers seemed to be related to the confiscation of their land for irrigation projects, and the uneven distribution of benefits among the agricultural sectors tended to skew toward large-scale agribusiness (Skidmore and Wilson 2007; McCarthy 2000; Hudson-Rodd and Htay 2008).

As the largest city in Myanmar, Yangon has been the epicenter of the nation’s socioeconomic and political transformations. However, due to the country’s isolation and the lack of data accessible to foreign scholars, few studies have succeeded in exploring urbanization in Myanmar, related spatiotemporal changes and the underlying socioeconomic and natural forces, except the followings. Nwe (1998) examined the demographic and area expansion of Yangon when three new towns were constructed in the late 1980s. Khaing (2015) described the demography of major cities in Myanmar and highlighted several major socioeconomic and environmental issues related to urban landscape changes. Myint (1998) studied the urban growth of Yangon and found that the expansion in early 1990s was largely due to the establishment of many new towns. The newly established town of Dagon alone was larger than the whole Yangon in the 1980s. Wang et al. (2018) evaluated the urban expansions of Yangon and the new capital city (Nay Pyi Taw) and their environmental consequences with five variables obtained from remote sensing data, including land surface temperature (LST), percent tree cover (PTC), evapotranspiration (ET), terrestrial ecosystem net primary productivity (NPP), and aerosol optical depth (AOD) from 2000 to 2013.

While being informative, these studies do not provide a comprehensive analysis of spatiotemporal change of cities in Myanmar, their major urban environmental impacts, and the major drivers since the beginning of the economic liberalization. To that end, we used Yangon as a case to reveal the urbanization pattern, impacts, and drivers in a radically transitioned economy in the developing world. Although it may not be a typical city in Myanmar, Yangon is selected due to its extremely important economic position in Myanmar and dramatic urban and environmental transformations after economic liberalization. In addition, Yangon’s urban development may have been affected by some other forces, such as both the capital relocation from Yangon to Nay Pyi Taw in 2005 and the occurrence of the extreme climate event of Nargis in 2008, in addition to serving as the primary gateway connecting Myanmar with the outside world, thus making it an ideal case to evaluate the impact of these external influences. We aim to answer the following research questions related to urbanization and environmental changes:

- What spatial and temporal changes in urban development have occurred in Yangon during the last three decades?
- What major environmental changes has Yangon experienced?
- How have the urbanization, economic development, and environmental changes co-evolved over time?
- How have extreme climate events, globalization, and the capital’s relocation to Nay Pyi Taw affected urban transformation in Yangon?

This paper illustrates the spatial and temporal changes of urbanization and reveals major environmental challenges experienced by Yangon. In addition, we highlighted the co-evolved relationship among urbanization, economic development, and environmental changes. Our approaches of using both quantitative and qualitative analysis to understand the urbanization process of a mega city in a transitional economies have great implications for other similar research.

## Methods

### Study area

**Fig. 1:**
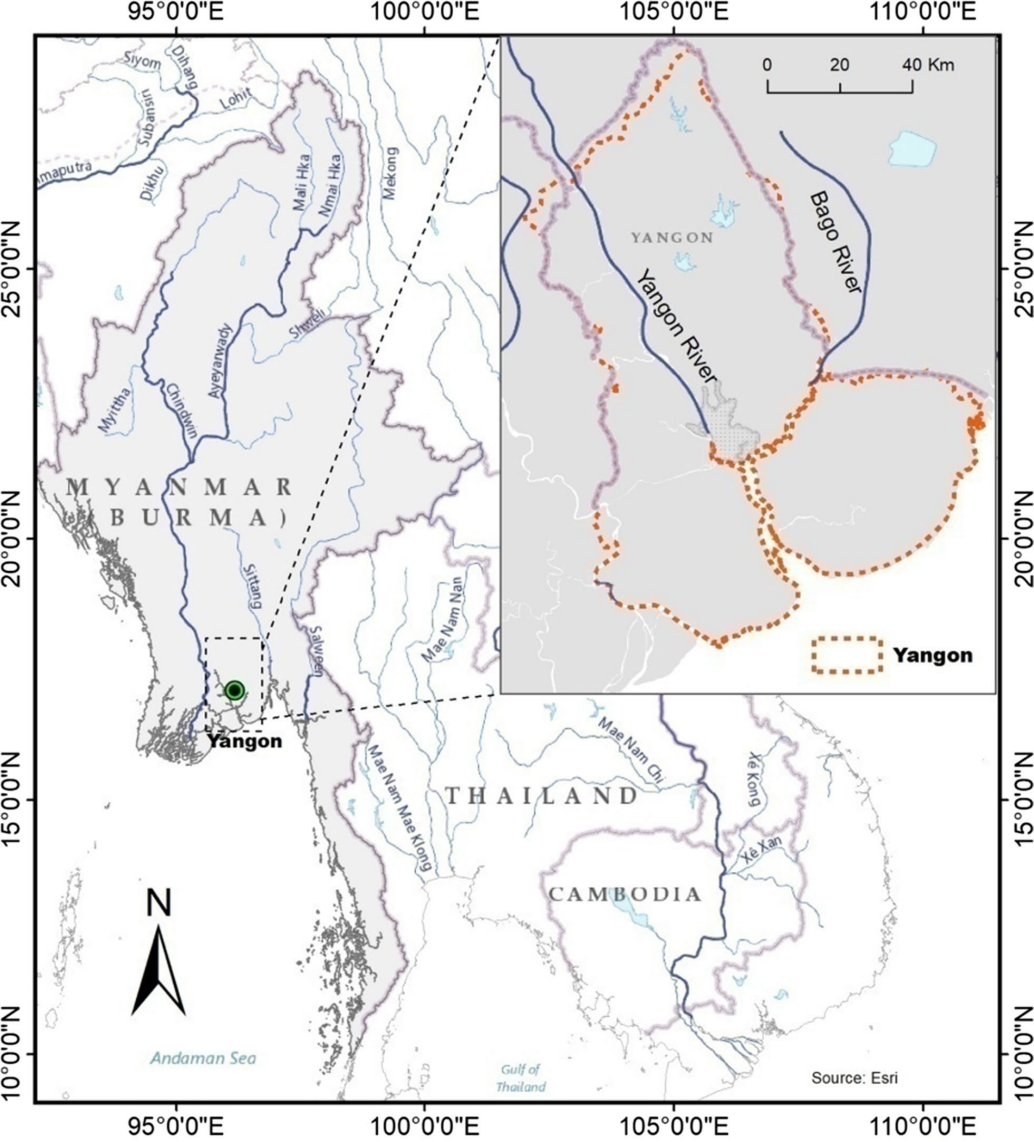
Location of the Yangon Region in Myanmar in Southeast Asia. The red-dotted lines mark the boundaries of the Yangon Region that is equivalent to a province or a state in many countries. The city of Yangon is located at the confluence of the Bago and Yangon rivers (blue lines)

Yangon (16° 51′ N, 96° 11′ E), formerly known as “Rangoon,” is located at the convergence of the Yangon and Bago rivers from the Gulf of Martaban (Fig. 1). It has a tropical monsoon climate with a rainy season from May through October and a dry season from November through April. The annual mean temperature is 27.4 °C (Myint 1998). With a metropolitan population of 7.4 million (5.2 million urban population) according to the 2014 census, it is the largest city in Myanmar and the economic and transportation center for the nation. Founded as a small fishing village in the eleventh century by the Mon people, Yangon did not have any significant urban development until the colonial period when the British turned the city into a commercial and political hub and later the capital for British Burma. The colonial city soon became known as “the garden city of the East” due to the spectacular urban landscape featured by parks and lakes, colonial buildings, traditional wooden architecture, and high-quality public infrastructure and urban services. Under the colonial government, Yangon expanded from 72.5 km^2^ in 1901 to 86.45 km^2^ in 1921 (Myint 1998). After Myanmar’s independence from the British in 1948, Yangon started to expand, with new satellite towns of South Okkalapa, North Okkalapa, and Tharkayta all built in 1958. Yangon has rapidly urbanized, since the country began its economic liberalization in 1988. Large new towns, such as Dagon, Hlaingtharyar, and Shwepyithar, as well as many small new towns, such as Weibergi, Shwepaukkan, Pale, and Padamyar, were established in 1988. The city expanded its area from 123 km^2^ in 1953 to 166 km^2^ in 1962, 209 km^2^ in 1974, 346 km^2^ in 1988, and 679 km^2^ in 1995 (Myint 1998).

### Data and analysis

We assessed the spatiotemporal changes of urban land expansion, the restructuring of urban land, and the environmental changes from for the last three to four decades based on satellite imagery, historic land use maps, and expert opinions. We analyzed the coupled dynamics among urbanization, economic development, and environmental changes of the city. We further discussed the influence of human systems (i.e., privatization, capital relocation, and globalization by FDI and tourism industry) and natural systems (e.g., extreme climate event of Cyclone Nargis) on Yangon’s urbanization and sustainability.

### Data processing

#### Urban built-up land data and processing

Land use data were classified from Landsat TM images in four periods (i.e., 1990, 2000, 2010, and 2020) to examine spatiotemporal changes. All Landsat TM images were downloaded from the United States Geological Survey (USGS) (https://www.usgs.gov/) Level-1 geo-referenced product, which were then converted to reflectance using the calibration function built-in ENVI 4.8. For each image, we classified it into five classes: urban built-up land, farmland, green land, water body, and bare land, using an object-oriented method as described in Ouyang et al. (2016), except that we manually identified bare land. For the detailed accuracy assessment method, please see Appendix 2.

#### Environmental data and processing

Due to lack of ground-station-based air pollution data, we derived surface air pollution data of fine particulate matter (PM_2.5_), NO_2_, and CO from 1997 through 2021 based on remote sensing products. Surface PM_2.5_ and NO_2_ mixing ratios were accessed from the Atmospheric Composition Analysis Group (https://sites.wustl.edu/acag/). The annual mean surface PM_2.5_ at 0.01 × 0.01 degrees resolution was estimated by combining aerosol optical depth retrievals from the NASA MODIS, MISR, and SeaWIFS instruments with the GEOS-Chem chemical transport model, and subsequently calibrated to global ground-based observations of PM_2.5_ using geographically weighted regression (van Donkelaar et al. 2016). The annual mean surface NO_2_ mixing ratio at 0.1 × 0.1 degrees resolution was also inferred from the GOME, SCIAMACHY, and GOME-2 satellite instruments (Lamsal et al. 2008). MOPITT data at 1 × 1 degrees resolution were accessed for processing surface CO mixing ratio (Deeter et al. 2003, 2013). We downloaded Version 6 Level 3 monthly CO data and then aggregated into annual means. To extract data for Yangon, we used the administrative area for the whole Yangon region and computed the spatial mean value. These data sets were obtained and exported from the Google Earth Engine data catalog and aggregated into annual means.

#### Socioeconomic and population data

We collected data on population and economic development, such as gross domestic product (GDP), GDP per capita (GDPpc), percentages of primary, secondary, and tertiary industries of GDP. The major sources of the demographic and socioeconomic data are from the Census of Myanmar and the Statistical Yearbook of Myanmar (Myanmar Government 2011, 2015). We also collected transportation-related data of Yangon, such as on number of vehicles and average speeds of private cars and buses at peak hours (Myanmar Government 2015). To understand the economic development and institutional mechanisms, we collected reports on topics such as the development of the industrial zones, documents, and news related to land regulation and urban development/planning policies.

#### Analysis on co-evolved relationships between urbanization, economic development, and environmental changes

We studied the co-evolved relationships between urbanization, economic development (measured by GDPpc) and environmental changes (measured by concentration of three air pollutants) by correlations and regressions. We complemented the quantitative analysis with qualitative methods of interviews and an expert panel. We have conducted multiple field trips to Yangon and interviewed eight local experts in urban development to understand major socioeconomic drivers and impacts of extreme events, globalization, and capital relocation on urbanization, economic development, and environment changes. We also organized a 1-day workshop on Yangon’s urban development with additional 20 local experts from Myanmar, mostly based on Yangon, in summer 2015. The experts are government officers, planners, and university professors in urban planning, economic development, and environment. They were divided into three groups and each group identified developmental stages and drivers of urbanization of Yangon after 1988.

### Extreme event, globalization, and capital relocation as drivers for urbanization

To understand the impact of the extreme event of Cyclone Nargis in 2008, we derived a map of flooded croplands and examined the migration from severely impacted areas to Yangon. We used the Moderate Resolution Imaging Spectroradiometer (MODIS) products to help identify and delineate flooded croplands during Cyclone Nargis given required data quality, temporal frequency and spatial resolution. Original data set was preprocessed using MRTools and ArcGIS for Desktop, including format conversion, reprojection (WGS84), study area extraction and reflectance calculation. Cloud mask was created to mitigate effect of cloud contamination after Nargis hit the low-lying Irrawaddy River Delta. Usually, cloud has a high reflectance in blue band compared to other objects, thus we could define an appropriate threshold to help mask out such atmosphere contamination. In this case, pixels with a blue band reflectance higher than 0.2 was classified into cloud. In addition, this threshold was in well with a previous study (Xiao et al. 2006). Meanwhile, we depicted croplands distribution over the entire delta using land cover classification information in an annual MOD12Q1 layer. The Normalized Difference Vegetation Index (NDVI) is a uniformed indicator for observing, measuring and understanding terrestrial vegetation activities from space. In addition, the Enhanced Vegetation Index (EVI) is designed to optimize vegetation signals, especially in high biomass regions, and improve vegetation monitoring through a de-coupling of the canopy background signal and atmosphere inferences (Huete et al. 2002). Both indicators can be employed to detect and investigate seasonal changes in vegetation growth. Here, we applied them to determine and evaluate pixels, where Nargis devastated croplands. Late April and early May is a good time for harvesting paddy rice in the delta; consequently, both NDVI and EVI values remain high in Yangon, Myaungmya, Pyapon and surrounding neighbors. However, heavy rainfall, strong winds and high tide surges brought on by Nargis flooded and destroyed mature crops and caused both vegetation indices to decrease sharply. Therefore, we assumed that any cropland pixel with lower NDVI and EVI values on May 5, 2008 after Nargis left the crop-rich regions were victims of the disaster. To understand the degree of globalization of Yangon, we collected data on topics, such as foreign direct investment (FDI), overseas development aid (ODA), foreign passengers and freight passage through Yangon. To evaluate the impacts of the capital relocation, we collected data on population in the resettlement area of Nay Pyi Taw and the pre-existing towns around Nay Pyi Taw from Department of Human Settlement and Housing Development (DHSHD), as well as compared the population of Yangon before and after the relocation.

## Results

### Urban expansion and urban transformation

**Fig. 2:**
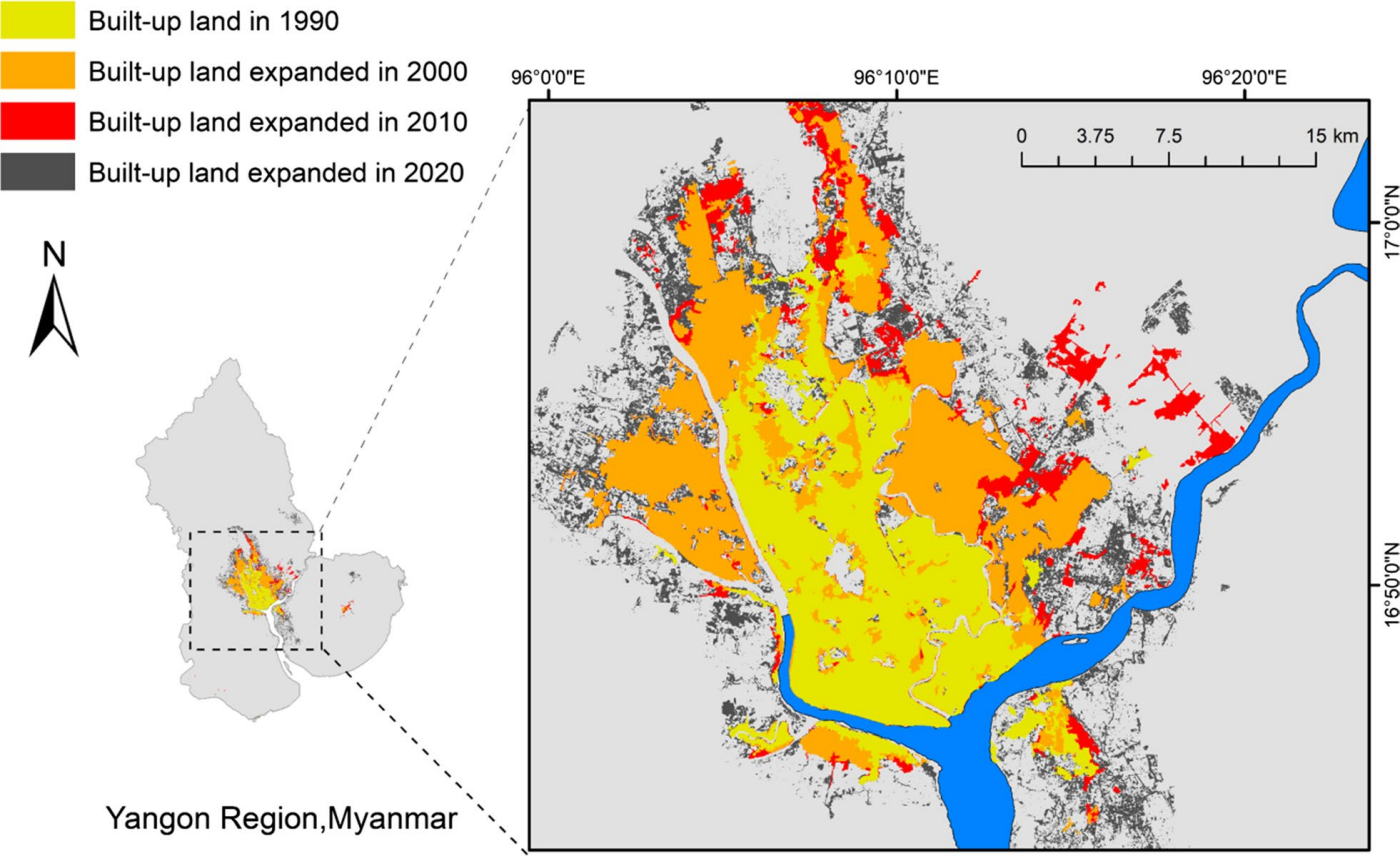
Urban expansion of Yangon, Myanmar during 1990–2020. The urban built-up area expanded from 161 km^2^ in 1990 to 289 km^2^ in 2000, 329 km^2^ in 2010, and 739 km^2^ in 2020

Yangon expanded its urban built-up land rapidly from 1990 to 2000, slowed down from 2000 to 2010, and gained momentum again during 2010–2020 (Fig. 2). The urban built-up area increased by 79% from 161 km^2^ in 1990 to 289 km^2^ in 2000, 104% to 329 km^2^ in 2010, and 225% to 739 km^2^ in 2020. Urban built-up land was mostly converted from farmlands and green land in both 1990–2000 and 2010–2020 (Table 1). In fact, 128 km^2^ (2%) of farmland was converted to urban built-up area during 1990–2000, contributing to 44% of urban land in 2000. During this period, the majority of new urban land expanded into the northeast/northwest, whereas little change was observed to the south of the Yangon River. Although a new town was established in the southern part of Yangon (i.e., Dala Township), urban land developed slowly due to the difficulties of water supply and commuting problems as there is no bridge to cross the Yangon River. Urban expansion continued into 2020 with an additional 328 km^2^ of farmland and 112 km^2^ of green land being converted to urban built-up land for the period of 2010–2020, more than doubling urban built-up area.

**Table 1:** Land conversion matrix (km^2^) for Yangon for 1990–2000, 2000–2010, and 2010–2020

	Land use	Water	Green land	Farmland	Urban built-up land	Bare land	Total
		1990					
2000	Water	*38*	87	88	7	0	220
	Green land	43	*1777*	474	27	1	2322
	Farmland	96	815	*5694*	10	0	6615
	Urban built-up land	15	29	128	*117*	0	289
	Bare land	0	2	1	0	*0*	3
	Total	193	2709	6385	161	1	9449
		2000					
2010	Water	*190*	43	47	2	0	281
	Green land	9	*2022*	157	9	1	2198
	Farmland	19	245	*6365*	9	1	6640
	Urban built-up land	2	11	47	*270*	0	329
	Bare land	0	0	0	0	*0*	1
	Total	220	2321	6615	289	3	9449
		2010					
2020	Water	*153*	12	195	3	0.0	363
	Green land	54	*1518*	168	14	0.4	1745
	Farmland	69	556	*5949*	18	0.2	6592
	Urban built-up land	5	112	328	*294*	0.0	739
	Bare land	0	0	0.2	0	*0.2*	0
	Total	281	2198	6640	329	1	9449

The number in each cell indicates how much (A) type of land in Year I (column heading) was converted to (B) type of land in Year II (row heading). For instance, of the cell (1990 Farmland, 2000 Urban land), the number is 128, which means 128 km^2^ of land was converted from farmland to urban area from 1990 to 2000

### Environmental changes

**Fig. 3:**
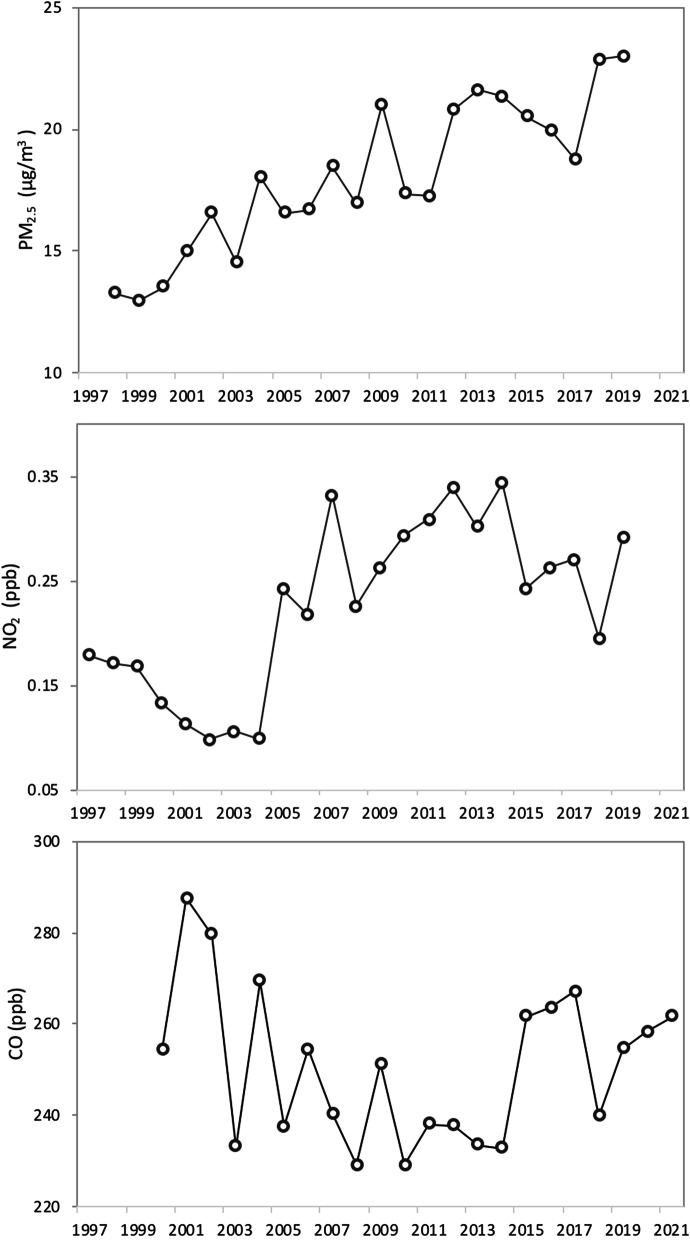
Changes in PM_2.5_, NO_2_, and CO concentrations in Yangon from 1997 to 2021. While the concentrations of CO decreased from the late 1990s, the concentrations of NO_2_ and PM_2.5_ increased from the late 1990s to 2019

We used three air pollutant concentrations to illustrate the changes in environmental conditions in Yangon (Fig. 3). While the concentrations of CO generally decreased over the years, from 287 ppb in 2001 to 262 ppb in 2021, the concentrations of NO_2_ increased from 0.18 ppb to 2.29 ppb and the concentration of PM_2.5_ had steadily increased from 13 μg/m^3^ in 1998 to 23 μg/m^3^ in 2019. PM_2.5_ of the whole period exceeds the annual mean of PM_2.5_ set by the National Ambient Air Quality Standards (NAAQS) of the United States Environmental Protection Agency (USEPA) at 12 μg/m^3^, but annual mean NO_2_ appeared lower than the NAAQS of 53 ppb. As for the CO concentration, the maximum concentration of 287 ppb (0.287 ppm) is above the global background concentration of CO (0.05–0.12 ppm) (WHO 2000).

### Coupled changes with economic development

**Fig. 4:**
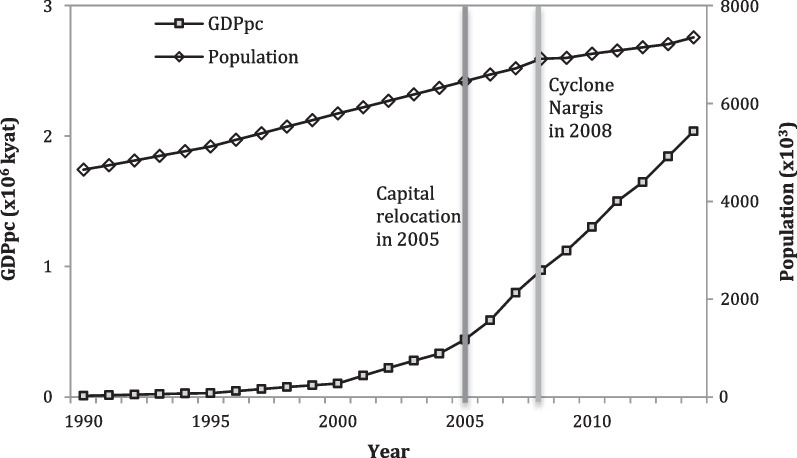
Changes in population and GDP per capita (GDPpc) in Yangon from 1990 to 2014. Note: Two external events and the growth of population and GDPpc of Yangon, capital relocation in 2005 and Cyclone Nargis in 2008. These two events seem do not have obvious impacts on city’s population or economic development level

**Fig. 5:**
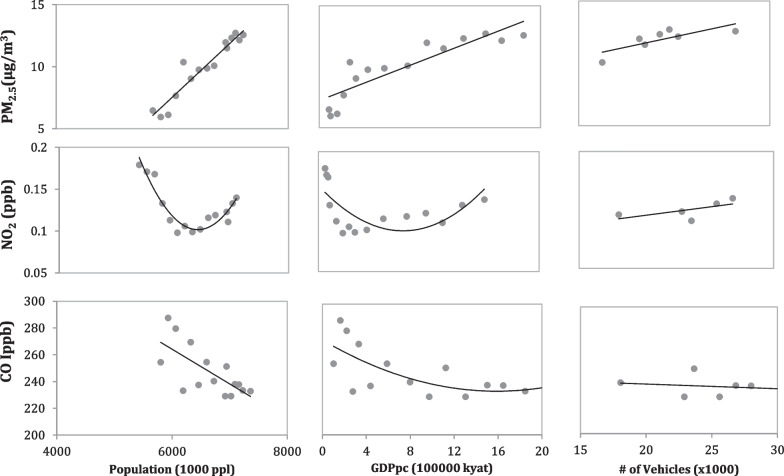
Changes in concentrations of three air pollutants (PM_2.5_, NO_2_, and CO) with three socioeconomic variables (population, GDPpc, and # of vehicles) in Yangon. PM_2.5_ was positively associated with all three socioeconomic variables; NO_2_ decreased and increased with population and GDPpc, respectively; and CO declined with population and GDPpc

Based on the data analysis from 1997 to 2014, Tthe empirical relationship between population and socioeconomic development is apparent (Fig. 4), with a correlation coefficient of 0.89 between them (Table 2). PM_2.5_ was positively correlated with population and GDPpc, with correlation coefficient of 0.95 and 0.89, respectively (Table 2, Fig. 5). In contrast, NO_2_ and CO had negative correlations with population and economic development (− 0.65 to − 0.19), showing a decline with population increase and economy development. It is worth noting that the number of vehicles has very high correlation with population and GDPpc (0.97 and 0.97, respectively), with PM_2.5_ and NO_2_ (0.78 and 0.64, respectively), but a negative correlation with CO (− 0.32). While automobile emissions have been recognized as one of the major sources for all three of these air pollutants, our analysis shows that the increase in vehicle number is only associated with increasing PM_2.5_ for Yangon (Table 2).

**Table 2:** Correlation (*r*) matrix among the six environmental and socioeconomic variables in Yangon based on data from 1990 to 2014

	PM_2.5_	NO_2_	CO	Population	GDPpc	# Vehicles
PM_2.5_	1	− 0.08	− 0.79	0.95	0.89	0.78
NO_2_		1	− 0.46	− 0.50	− 0.19	0.64
CO			1	− 0.65	− 0.63	− 0.32
Population				1	0.89	0.97
GDPpc					1	0.97
# Vehicles						1

All correlations are significant with *p* values < 0.001

### Urban development stages identified by the workshop

In addition to interviews, we organized a 1-day workshop with 20 local experts in summer 2015 to understand the urban development of Yangon. Expert discussions in our workshop highlighted different sets of drivers of urbanization in Yangon in three distinct periods after 1988, i.e., 1988–1992, 1992–2005, and 2005–2015. It should be noted that these periods do not correspond to the land use data we used to analyze urban expansion of Yangon for 1990, 2000, 2010, and 2020. Rather, urban development in these three periods were driven by different major forces. For the first period (1988–1992), the policies for the construction of new towns played a major role in the urban expansion of the city, resulting in the construction of a new town area that was equivalent to the city area in 1983. In 1988, the military government took over the socialist government and implemented urban development policies focusing on new town development, including New Dagon, Mingalardon, and Shwe Pauk Kan northeast of the inner city, and Shwe Pyi Thar and Hlaing Thar Yar northwest of the inner city.

For the second period (1992–2005), the private sector and capital markets gradually became the major forces for development. Land speculation started in 1991 as people lost trust in the strength of local currency and felt safer investing in land. The city expanded without any urban planning or proper urban infrastructure development. As a result, many low standard and unhygienic areas were developed lacking proper drainage, sewage, and garbage collection systems.

For the third period (2005–2015), the private sector continued playing a strong role but three new major drivers appeared: (1) relocation of the capital from Yangon to Nay Pyi Taw in 2005, (2) the Cyclone Nargis in 2008, which caused a large inflow of migrants to Yangon, and (3) elevated foreign investment, especially in the real estate market, after Myanmar transitioned from military to civilian government in 2010. For example, a Vietnam-based real estate developer constructed the Myanmar Plaza, located in the inner city near Inya Lake. Several Chinese development firms also invested in the real estate in Yangon and Mandalay, starting a new wave of city building in Yangon. In 2015, the National League for Democracy (NLD), led by Aung San Suu Kyi, won the majority of the seats in both chambers of the national parliament, leading to the change from the “civilian government” (note that ~ 90% of the government cabinet members were ex-military officers) to the democratic government. Therefore, the momentum further escalated as more large-scale real estate development projects appeared.

The workshop also identified four major environmental challenges: (1) urban service provision and distribution, (2) traffic congestion, (3) urban flooding, and (4) lack of green space. Yangon had faced mounting pressure to improve urban service provision and distribution, especially on garbage collection, drainage systems, and water supply. The city generated ~ 1600 tons/day of garbage for its landfill facility. However, some outer areas lacked services for garbage collection and sewage management. Amongst many consequences was the blockage of drainage systems. Relying primarily on four surrounding reservoirs and underground water, Yangon has the capacity to supply 160 million gallons of water to its 5.2 million inhabitants (i.e., 30 gallon per person per day). However, only 65% of Yangon’s population was served, whereas four towns (Dagon Seikan, Hlaing Thar Yar, Shwe Pyi Thar, and Seikkyi-Kanaungto) are poorly served. Due to leakage and accounting problems, it is estimated that more than 30% of water is either lost to leakage or not otherwise accounted. This challenge in urban service provisioning reflects the thesis of urban environmental transition (McGranahan et al. 1996), which states that for cities with low economic development, environmental actions should focus on local scale and intermediate issues, such as garbage collection, wastewater, and water supply.

**Fig. 6:**
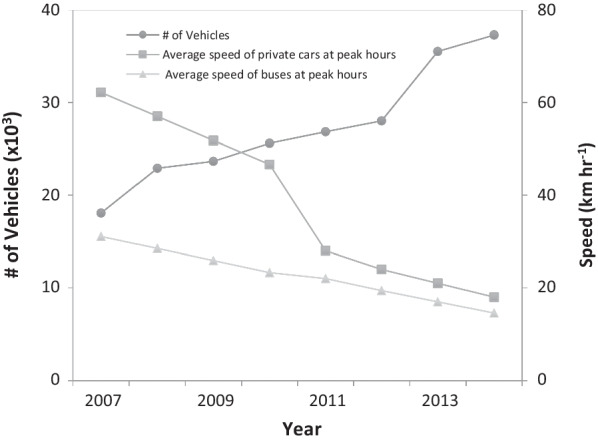
Number of vehicles and average speed for private cars and buses at peak hours in Yangon, Myanmar. The number of vehicles doubled from 2007 to 2014. Meanwhile, average speed of buses and private cars at peak hours both decrease dramatically, leading to the speeds decreased to less than 1/2 or 1/3 of the 2007’s levels for buses and private cars

Yangon has faced serious traffic jams due to a combination of rapid population growth, poorly designed road network, increased wealth, the relaxed policies on importation of vehicles. Since 2010, the government has allowed the importation of cars without much restriction, leading to a significant increase in private vehicles (Fig. 6). From 2007 to 2014, the number of vehicles in Yangon doubled within 7 years, from around 18,000 in 2007 to more than 37,000 in 2014. The total number of vehicles increased quickly, whereas the average speed of either private cars or buses at peak travel hours decreased accordingly. Average speed of private cars and buses both declined, with private cars decreasing more dramatically in 2010 when the restriction on car imports was lifted. The average speed of private vehicles in peak hours was only 18 km/h in 2014, less than one-third of the speed in 2007 (62 km/h) in 2007.

Flooding represents another serious challenge because of the geophysical and natural setting of Yangon, i.e., located in the river delta and in the Asian monsoon region. Due to the lack of adequate urban infrastructure including drainage systems, and the blockages from uncollected garbage mentioned previously, Yangon easily and frequently floods during the rainy seasons. Because of the serious repercussions of urban flooding, the city paid 1 million USD in the 2016–2017 fiscal year to private contractors to drain storm water. Moreover, Yangon’s mayor announced in 2017 that the city would use US $77 million, a significant part of the US $124 million that World Bank loan intended for public infrastructure, to fund projects to end the perennial flooding problems in Yangon (Win 2017).

Green space provision was identified as a major urban environmental problem and one that has been a contentious topic in Yangon. While the colonial city of Yangon has been praised as “the garden of the East” due in part to its beautiful lakes and park spaces, the expanded city under the socialist era did not consider green space in their urban designs and developments, except through the allocation of small portions of the land at the corners of a neighborhood as playgrounds or sport fields. This resulted in extremely low coverage of green space (38%) and low green space per capita (21 m^2^), compared with other regional cities in Southeast Asia (48% and 68 m^2^) (Richards et al. 2017). More importantly, green space was further reduced when the military-controlled government allowed commercial development on these already limited public spaces. Despite protests from the residents, much land in and around parks and lakes has been auctioned away for commercial development in the past two decades. When enough in-situ ground data can be collected, further research can be conducted on how green space may affect the spatiotemporal changes in air pollution in tropical urban climates, such as Yangon, as illustrated by research on other cities with sub-tropical climates in Asia (Chen et al. 2016).

## Discussion

### Economic and environmental changes during urbanization

Urbanization, economic development, and environmental changes have complex relationships and pose great challenges for Yangon (Table 2, Figs. 4 and 5). It was not surprising to see the strong correlation between population and economic development, although it is interesting to witness population coupled with rapid growth of GDPpc (Fig. 4). This implies that the productivity gain due to the agglomeration effects of the economy may outweigh the downsides of increased population concentration, such as congestion (Batty 2008). As for the relationships between air pollution and GDPpc, while the concentration of PM_2.5_ increased as population increased and the economy developed (Fig. 5), the concentrations of two other air pollutants (NO_2_ and CO), especially NO_2_, did not have strong correlations with either population or economic development (Table 2). Indeed, the trend lines indicated a “U” relationship between NO_2_ concentration and population or economic development. As Yangon has not yet transformed into an industrial city, we suspect that the strong relationships between air pollutants and economic development at the early stage of industrialization may not be apparent. A similar relationship had been reported in Ulaanbaatar and large cities in Vietnam (Fan et al. 2016, 2019; Fan 2022). Furthermore, the differing relationship between air pollutants and the number of vehicles not only implies the multi-source nature of air pollution but also illustrates that industrial emissions may not be as important as automobile emissions as the major source of air pollution in Yangon. Our findings have highlighted the needs for a more nuanced understanding of drivers of urbanization and environmental changes in the developing world.

### Major external drivers not captured by current data analysis

Although our expert panelists highlighted the importance of the capital relocation, the extreme event of Cyclone Nargis, and globalization as external drivers for the most recent period of urban development in Yangon, we were not able to detect their impacts from the changes in demography and economic development (Fig. 4). No significant changes in the population and GDPpc were found for 2005 or 2009. This phenomenon highlighted the need to place our study in the context of locals through obtaining opinions of local experts. Due to the insignificant amount of relocated population and the inability of obtaining official statistics on rural–urban migrants, the impacts of this extreme event and the capital relocations were not reflected in the statistical data of the population. Thus, relying only on what seems-to-be official data can be misleading. Similarly, the impact of globalization is complex and hard to measure. More efforts are needed to untangle the impacts of these external drivers (see Appendix 1 for details).

### Limitations and future research

While our research is limited mainly due to the unavailability and unreliability of official statistics, we partially resolved the issue through using other sources such as satellite images to derive urban land and air quality data. However, it is beyond a single research team’s ability to collect long-term, large-scale socioeconomic data. With the current unstable political regime in Myanmar, this remains a challenge for any comprehensive scientific investigations on the city, as well as the country. Myanmar’s first census was conducted in 2014 with technical advice and funding from international organizations and donors. However, it has received many criticisms since then. Other data issues include the impact on our analysis due to an artificial foreign currency exchange rate. For example, using the official statistics, we found that FDI as a percentage of GDP remains low (< 1%), indicating that Myanmar’s economy is still quite closed-off from the global economic system. However, a closer look by correcting the artificial exchange rate to the real exchange rate illustrated that FDI in Myanmar is quite variable, with FDI as a percentage of GDP ranging normally between 1% and 3%, but with some high peaks of 17% in 1997, 25% in 2005, and 50% in 2010.

Our expert panel highlighted a distinctly different set of forcing mechanisms that had affected the urbanization processes for 1988–1992, 1992–2005, and 2005–2015, as well as for the major environmental challenges of the city: urban service provision, traffic congestion, flooding, and green space. The expert panel identified the capital relocation, Cyclone Nargis, and globalization as the three most significant drivers of urbanization in the most recent period of urban development. However, their impacts cannot be validated through the official demographic or economic data. Clearly, to conduct a critical analysis, future attention needs to be placed on this mismatch among official data, remote sensing, and the reflections of local experts. Currently, the spatial resolution of air pollution (0.01°, ~ 1.11 km) is too coarse to conduct a meaningful spatial analysis on air pollutants with urban built-up land use data which has a spatial resolution of 30 m. In future, if in-situ observation data from local air quality monitor stations are available, more fine scale (currently air pollution map has 0.01 degree resolution, about 1.1 km) spatial maps of three air pollutant concentrations at higher resolution may also be generated. The spatial analysis about the relationship between the concentration of different air pollutants and urban built-up land (30 m resolution) can be conducted to reveal how urbanization has affected patterns of air pollution.

### Implications of the research

Myanmar’s isolation from international investment and its weakened economy from sanctions following a 1988 military coup further exacerbated the country’s economy. While the transition to civilian administration in 2011 provoked hopes of positive transformations, the military launched another horrible coup in 2021, arresting opposition leaders, including de facto leader Aung San Suu Kyi, civil society activists, and officially elected members of the congress (Thein and Gillan 2021). These difficult social, ecological, and political situations make the use of remote sensing a critical tool for tracking land use and changes. In combination with a human–environment systems approach and other secondary socio-economic data, this study investigated the complexity and dynamics of land change system transitions, and quantified the economic value of key services in connection to air pollution. It can be expected that there will be an extraordinary level of economic and social crises and an exceptional uncertainty in the country’s near future.

## Conclusions

We used Yangon as a case to reveal the urbanization, environmental changes, and the underlying driving forces in a radically transitioned economy in the developing world. We assessed the spatiotemporal changes of urban land, the environmental changes in air pollutants, and major drivers of urbanization from satellite imagery, historic land use maps and expert panels. We found that the city expanded urban built-up land rapidly from 1990 to 2000, slowed down from 2000 to 2010, and regained momentum again for 2010–2020, with most newly added urban built-up lands appearing to be converted from farmland and green land in both 1990–2000 and 2010–2020. Furthermore, the air pollutant concentration of CO decreased, but that of NO_2_ and PM_2.5_ increased in recent years. We further analyzed the interdependent changes between urbanization, economic development, and environmental changes. A positive correlation exists between population and economic development. It is worth noting that the concentration of PM_2.5_ is highly associated with population, the economy, and the number of the vehicles. Our research illustrates how the complex relationships between urbanization, economic development, and environmental changes can be untangled and analyzed. It confirmed the strong correlation between urban population growth and economic development level indicated GDPpc. Our finding implies that industrial emissions may not be as important as automobile emissions as the major source of air pollution in Yangon. As Yangon has not yet transformed into an industrial city, the strong relationships between air pollutants and economic development at the early stage of industrialization may not be apparent. Finally, the expert panel also identified other potential drivers for urbanization, i.e., the extreme climate event of Cyclone Nargis, capital relocation, and globalization. However, the impacts of these drivers were not captured by any quantitative data sources. Our research thus underlies the need to combine both quantitative data derived from satellite images and official statistics and qualitative data provided local experts to conduct a critical analysis, especially for cities in the developing world. The most recent military takeover remains an uncertain driver for Yangon’s future, highlighting the use of remote sensing as a critical tool for tracking land use and changes and environmental changes in certain part of the developing world.

## Figures and Tables

**Fig. 7:**
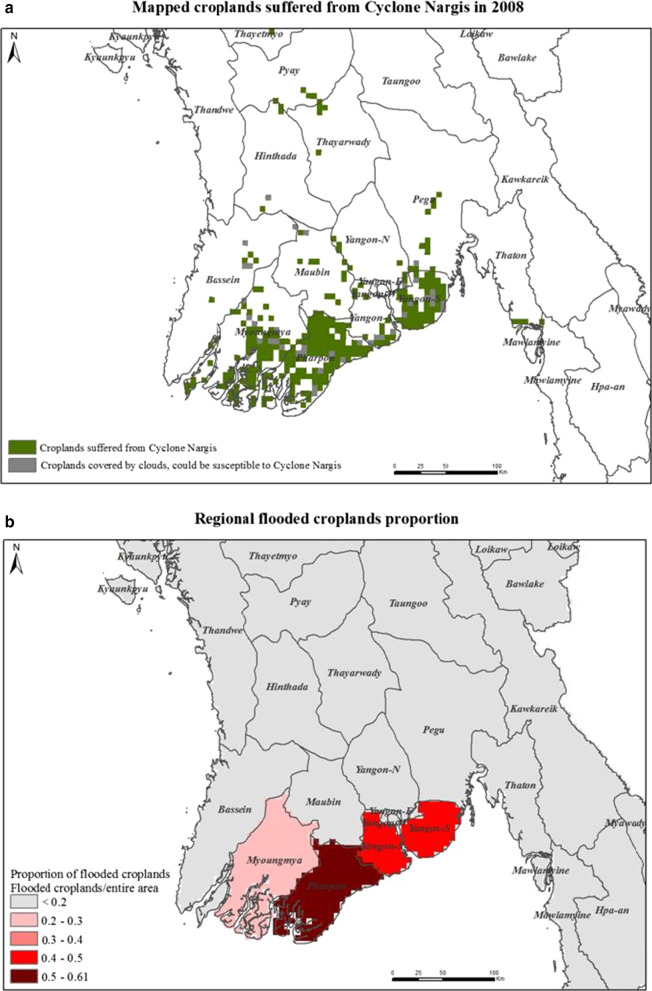
Impact of the extreme event—Cyclone Nargis: A1a (top figure). Croplands suffered from Cyclone Nargis (2008) in the low-lying delta of Myanmar; A1b (bottom figure) Regional proportion of flooded croplands

**Fig. 8:**
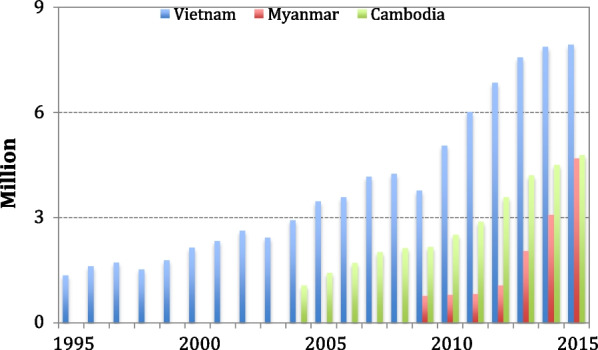
Foreign tourists in Vietnam, Myanmar, and Cambodia (1995–2015). Myanmar’s tourists increased rapidly in the early 2010s, but it is still far behind its neighboring countries of Vietnam and Cambodia

## Data Availability

The data sets used and/or analyzed during the current study are available from the corresponding author on reasonable request.

## Appendices

### Appendix 1. Major external drivers un-captured by current data analysis

#### Extreme event of Cyclone Nargis

The changes in global climate have profoundly affected Monsoon Asia, such as more frequent floods and intense droughts on agricultural and aquatic ecosystems that directly affect the livelihood of farmers, particularly the “subsistence” or “smallholder” farmers (e.g., Chotamonsak et al. 2011; Morton 2007; Zhai and Zhuang 2012). Unfortunately, the literature is scant on the effects of extreme climate events on urban processes, even though they are considered important drivers of urbanization. For example, urban processes can provide a push for rural–urban migration. Cyclone Nargis on May 2, 2008 was the worst natural disaster in the recorded history of Myanmar when at least 138,000 causalities were reported. The Yangon Region and the Ayeyarwady Region were affected by Cyclone Nargis, with a total damage estimate of at least US $10 billion (Toronto Star 2008). They were also the two most affected regions in terms of flooded croplands (Fig. 7). The aftermath caused a huge rural-to-urban migration from the rural areas of both states to Yangon for urban-related employment. An anecdotal estimate showed that > 90% of the rural–urban migrants who work in the garment or construction sectors are from Ayeyarwady. Many of these migrants lost their rural livelihoods due to Cyclone Nargis. These migrant workers usually settled down in informal settlements in the southern part of Yangon, where poor services were provided by the city. Unfortunately, official statistics on migration following Cyclone Nargis do not exist. Thus, although our experts have identified the extreme event as a significant driver for the rural–urban migration, no official statistics can provide such evidence.

Yangon, Pyapon and Myaungmya suffered the most from a huge agricultural economic loss due to Cyclone Nargis, with a total area of 73.74 km^2^ of affected croplands. Around 61% of the entire Pyapon experienced a paddy rice loss, followed by 41% in southern Yangon and 28% in Myanmar (Fig. 7b).

#### Globalization

Globalization, the international integration process from economic, cultural, and political influences, and information and technology, has affected the development of various regions including transitional economies, particularly through economic globalization—the international integration of the world production system through trade, investment, and production (IMF 2000; Sassen, 1991). In urban studies, globalization has been identified as an important driver causing divergent development of cities based on their positions in the hierarchical networks of world cities, but affected by different kinds of flows of capital, technology, and labor (Beaverstock et al. 1999; Castells 2011; Sassen 1999). Recent studies have also examined how globalization has affected urbanization and urban environments in transitional economies (e.g., Fan et al. 2017a, b). Relevant data for Yangon are unfortunately not available; thus, we used data for Myanmar, including trade, foreign direct investment (FDI), and international tourists, as proxies to assess how Yangon is connected to the global economy. Myanmar was well integrated with the world economy, as indicated by trade, foreign direct investment, and visits by foreign tourists. For example, 20% of the national GDP was from trade during the 1990s and the 2000s, with exports increasing more than 18 times from US $476.5 million in 1990 to US $8861.0 million in 2010, and from US $888.6 million in 1990 to US $6412.7 million in 2010 (i.e., an increase of more than 7 times). FDI also increased significantly in Myanmar since 1990 with the number of foreign companies and branches increasing by 15 times from 82 in 1990 to 1283 in 2010. Myanmar steadily increased its foreign tourism and started to catch up with its neighbors in Mainland Southeast Asia. However, political instability still presents a major threat to foreign direct investment and tourism, especially when comparing Myanmar with its neighboring countries.

As the largest city in Myanmar, Yangon was exposed to the force of globalization as early as 1853 when it became the colonial capital of British Burma, well as the whole country was largely shunned by the rest of the world, particularly beginning from the inception of military rule in 1962. It was not until 2011 when the military junta dissolved itself and transitioned the power to the civil government that Myanmar started to open up to the world again. The brief period of reconnecting to the world in recent years, however, has been intense and diverse and has dramatically increased flows of trade, foreign investment, overseas aid, foreign tourism, among additional matters that were revealed by our findings. For example, while less than 0.8 million foreign tourists visited Myanmar in 2009, in 2015, it welcomed six times more for a total of 4.7 million foreign tourists, almost the same as Cambodia (4.8 million) (Fig. 8).

#### Capital relocation

In November 2005, Myanmar officially relocated its capital from Yangon to Nay Pyi Taw (NPT). Almost all the government agencies, along with their employees, moved to the new capital. It is estimated that over 500,000 population of NPT, 65,000 government workers of the ~ 80,000 total were relocated from Yangon to NPT. However, the population relocation from Yangon to NPT was not detrimental to Yangon as it only accounted for ~ 1% of Yangon’s population. Interestingly, these government employees did not really relocate in the sense that their families still resided in Yangon due to the superior education, medical and cultural amenities of the city; commuting is widely practiced between Yangon and NPT. The real impact of the capital relocation is that the buildings and land that used to belong to the government now were leased mostly for commercial development. Due to skyrocketing land prices in Yangon, the government can reap huge profits by leasing their buildings, which usually have high historic and cultural values, to the private sector, despite the protests from Yangon residents who advocated for preserving these valuable cultural assets. The impact of privatization of the land and buildings with high historic, environmental, and cultural values remains to be evaluated.

Capital relocation is not uncommon. Historically, developed nations, such as the United States, Canada, and Australia, have relocated their capitals to either a small town or constructed a new city for its national capital. Several countries from the developing world have also relocated their capitals in recent decades, such as Brazil (from Rio de Janeiro to Brasilia in 1961), Malawi (from Zomba to Lilongwe, initiated in 1975 and completed in 1994), Nigeria (from Lagos to Abuja in 1991) and most recently Myanmar (from Yangon to Nay Pyi Taw in 2006), etc. There are multiple reasons for these relocations, such as to balance different regional/ethnicity interests (US, Canada, Australia, and Nigeria), to enhance linkages with neighboring countries (Russia), to decongest the original capital (Nigeria, Kazakhstan), to stimulate development in underdeveloped regions (Cote d’Ivoire), or security concerns (Myanmar). Relocations unavoidably affect the original capital city and the new capital. Yet, limited literature has examined how external shocks such as capital relocation have affected both the source and destinations. Some evidence shows that the original capital cities may be not impacted, especially those large cities in either developed or developing nations due to the failure of the new capital to provide counter-attraction against the old capital such as in the case of Malawi (Potts 1985). It was also noted that strong government commitment can facilitate the transition. Although the rationale of the movement was that the central location of NPT may provide easy access to all parts of the country, other speculation has existed, including the fear of foreign attack and greater control over the ethnic minority of the border region. Capital relocation had significantly changed NPT’s urban development with the dramatically increased urban built-up land and population in either the planned or unplanned periphery zone of NPT. The relocated population settled in both new resettlement areas in Nay Pyi Taw and the three pre-existing towns around Nay Payi Taw, Pyinmana, Leway and Tatkon, which were incorporated within National Union Territory in 2011 (Table 3). In 2007, the total population density of the three pre-existing towns grew from 113 persons/ha in 2004 to 142 persons/ha in 2007 due to the relocation.

**Table 3:** Population changes in Nay Pyi Taw and surrounding area. Source: Department of Human Settlement and Housing Development (DHSHD), 2011

Resettlement in Nay Pyi Taw from 2003 to 2011					
Resettlement area	Area (ha) (net)	No. of land plots	Estimated population	Density (persons/ha)	Established year
Thapyaegone	407.52	977	4885	12	2003–2009
Shwenatha	232.29	1194	5970	26	2006–2009
Nyaung Pingyisu	210.85	472	2360	11	2007–2011
Shwekyarpin	1176.04	4221	21,105	18	2009–2011
Total resettlement	2026.70	6864	34,320	17	

Increase of population in 3 pre-existed towns around NPT 2004–2007					
Town (urban)	Area (ha)	2004		2007	
		Population	Density (persons/ha)	Population	Density (persons/ha)
Pyinmana	826	85,324	103	133,970	162
Hleway	355	34,000	96	51,222	144
Tatkone	433	66,000	139	51,706	119
Total	1614	185,324	113	236,897	142

### Appendix 2. Land classification accuracy assessment

We conducted an accuracy assessment by first creating a stratified random sample of 200 points for each of the urban land layers for 2010 and 2020. Each sample was verified using very high-resolution Google Earth imagery (TerraMetrics 2022), as well as Landsat 7/8 RGB imagery (30-m spatial resolution) for 2010 and 2020, respectively; the median across all images for each year (2010 and 2020) was used for the verification layers. Landsat 7/8 imagery is courtesy of the U.S. Geological Survey and was accessed and processed in Google Earth Engine (Gorelick et al. 2017). For 2010, the overall accuracy came to 76.0%, and for 2020, the overall accuracy came to 72.5%. The 2020 urban land classification was also compared to a recently distributed 10-m spatial resolution ESA WorldCover data set (Zanaga et al. 2021). This data set was resampled to 30-m spatial resolution to match the Landsat classifications. After resampling, the number of urban pixels within the study area was 588,237 (an area approximately 503 km^2^). Of the ESA WorldCover urban area, the Landsat-based classification used here covered approximately 56.9% of the area. Of the Landsat-based classification, 65.4% was also characterized as urban by the ESA WorldCover layer.

## Abbreviations

LST: Land surface temperature
PTC: Percent tree cover
ET: Evapotranspiration
NPP: Terrestrial ecosystem net primary productivity
AOD: Aerosol optical depth
USGS: United States Geological Survey
GDP: Gross domestic product
GDPpc: GDP per capita
MODIS: Moderate Resolution Imaging Spectroradiometer
NDVI: Normalized Difference Vegetation Index
EVI: Enhanced Vegetation Index
FDI: Foreign direct investment
ODA: Overseas development aid
DHSHD: Department of Human Settlement and Housing Development
NAAQS: National Ambient Air Quality Standards
USEPA: United States Environmental Protection Agency
NLD: National League for Democracy
